# Crystallographic Characteristic Effect of Cu Substrate on Serrated Cathode Dissolution in Cu/Sn–3.0Ag–0.5Cu/Cu Solder Joints during Electromigration

**DOI:** 10.3390/ma14102486

**Published:** 2021-05-11

**Authors:** Wu Yue, Chao Ding, Hongbo Qin, Chenggong Gong, Junxi Zhang

**Affiliations:** 1 School of Materials Engineering, Lanzhou Institute of Technology, Lanzhou 730050, China; gongcg@lzit.edu.cn (C.G.); zhangjx@lzit.edu.cn (J.Z.); 2 School of Mechanical and Electronic Engineering, Guilin University of Electronic Technology, Guilin 541004, China; 19012302007@guet.edu.cn

**Keywords:** electromigration, solder joint, cathode dissolution, crystallographic characteristic, Cu substrate

## Abstract

The crystallographic characteristic effect of Cu substrate on cathode dissolution behavior in line-type Cu/Sn–3.0Ag–0.5Cu (SAC305)/Cu solder joints during electromigration (EM) was investigated by scanning electron microscope (SEM), electron backscatter diffraction (EBSD), and first-principles calculations. The SEM and EBSD results show that the crystallographic characteristic of Cu substrate is crucial to cathode dissolution behavior under a direct current of 1.5 × 10^4^ A/cm^2^ at 125 °C ± 2 °C. When the (001) plane of copper grain adjacent to the Cu_3_Sn/Cu interface is perpendicular or nearly perpendicular to the current direction, local cathode dissolution tips are easily formed, whereas the (111) plane remains mostly undissolved, which finally leads to the inhomogeneous cathode serrated dissolution in the substrate. The first-principles calculation results reveal that the different surface energies and energy barriers of the different crystallographic planes of Cu grains in the substrate are responsible for the local cathode dissolution tips. Adjusting the copper grain in a substrate to a crystal plane or direction that is difficult to dissolve during EM is a promising method for improving the reliability of solder joints in the future.

## 1. Introduction

The increasing demand for miniaturized, multifunctional portable electronic products and devices has led to a continuous scaling-down of the dimensions of solder joints in microelectronic packaging. Such downscaling dramatically increases the current density in the solder joints, causing electromigration (EM), that is, the directional migration of atoms under a high-density electric current [1,2,3,4]. EM can significantly deteriorate the performance of the solder joints, such as the polarity growth of the interfacial intermetallic compound (IMC), the ductile-to-brittle transition of the solder matrix, the hillocks or whiskers, the cathodic crack, and even the direct opening failure [5,6,7,8,9,10], etc.; therefore, it has become a serious reliability concern in microelectronic devices and products. Additionally, the high current density also brings the Joule effect and thermomigration (TM), which can significantly enhance the local temperature of solder joints and even lead to the liquid–solid migration [11,12]. Meanwhile, thermomechanical stress can be induced by the couple of EM and TM [13]. Hence, EM-induced failure has been widely researched. The failure mechanisms of solder joints induced by EM are extensively studied and many results have been obtained [14,15,16,17], and one of the most well-known results is that when the *c*-axis of *β*-Sn grains, which usually refers to the Sn grain orientation, is aligned or nearly aligned along the current direction, the Cu atoms diffuse quickly and the EM deterioration is more serious [18,19].

Recently, it has been reported that the current density over 5 × 10^3^ A/cm^2^ may induce the cathode dissolution of the interfacial IMC and Cu substrate, and some investigations reveal that the dissolution of the IMC is very complicated and closely related to two factors: the anisotropy of *β*-Sn grains and the unstable grain boundary grooves between interfacial Cu_6_Sn_5_ grains [15,20,21,22,23,24]. When the *c*-axis of *β*-Sn grains is aligned or near-aligned to the current direction in line-type Cu/Sn/Cu interconnects, the diffusion of Cu atoms is fast and, consequently, the IMC dissolution becomes more severe [23]. This has also been proven in a previous study looking at the IMC growth in idealized single-crystal joints using Sn–3.0Ag–0.5Cu (SAC305) solder alloy [13]. Additionally, the rapid diffusion of Cu atoms at the boundary groove also leads to the nonuniform consumption of Cu substrate and the serrated cathode interface [15,20]. Some studies further revealed that the *c* axis of Sn grain dominated the dissolution of Cu substrate (or Ni substrate) and the second phase Cu_6_Sn_5_ during EM [18,19,25,26,27]. These results reveal that the reliability issues of solder joints are significantly dominated by the anisotropy of *β*-Sn grains during EM. It seems that the EM deterioration of solder joint induced by EM has nothing to do with the crystalline characteristic of the substrate. As an essential component of the solder joint, the crystallographic characteristic of the cathodic substrate may have an important effect on its own dissolution and the reliability of solder joints. However, probably, due to the isotropy of the substrate material polycrystalline copper, few literatures have investigated the crystallographic characteristic effect of Cu grains on the cathode dissolution during EM. In fact, the crystallographic orientation of copper grains and grain boundaries do play important roles in the evolution of voids in dual-inlaid copper interconnects during EM [28]. Therefore, it is of engineering significance to study the effect of crystalline characteristic of Cu substrate on the cathode dissolution behavior of solder joints during EM.

In this study, the crystallographic characteristic effect of Cu substrate on the cathode dissolution behavior in line-type Cu/SAC305/Cu solder joints during electromigration (EM) was investigated by scanning electron microscope (SEM), electron backscatter diffraction (EBSD), and first-principles calculations.

## 2. Materials and Methods

**Figure 1:**
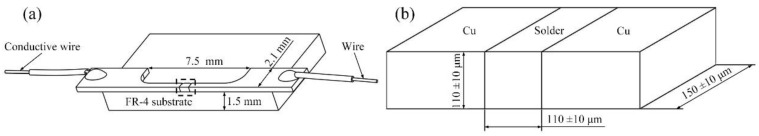
Schematic of the experiment configuration (**a**) and the final dimension of the solder joint (**b**) marked by the dash-line in (**a**).

The line-type Cu/SAC305/Cu solder joints were fabricated by the following procedure. Firstly, the commercial polycrystalline copper foil (99.9 wt%) with a thickness of 150 μm was cut into strips with a width of 8 mm and length of 20 mm, and the ends of the strips were mechanically ground with waterproof sandpapers of different grits in succession and polished with Al_2_O_3_ suspension liquid (in which the diameter of Al_2_O_3_ particle was 50 nm). Then, the strips were ultrasonically cleaned in ethanol. Afterwards, the solder joints were assembled and prepared using SAC305 solder on a specially designed fixture by modeling the reflow soldering process with a peak temperature of 280 °C. When the solder was completely melting and the solder interconnects were formed, the samples were kept for 80 s and then cooled in air. Finally, the samples were mechanically machined to the desired U-shape geometry, soldered the wire, fixed onto the epoxy glass cloth board using a commercial instant adhesive with an adhesive thickness of about 1 μm, and then ground and polished to the final dimension, as shown in Figure 1. Other details refer to our previous study [17].

In the EM test, the solder joint samples were stressed by a direct current of 1.5 × 10^4^ A/cm^2^ at 125 ± 2 °C. The interfacial evolution of the joints after 200 h under EM was investigated by SEM (Zeiss Supra 55, Carl Zeiss Co., LTD, Aalen, Germany) and EBSD analysis, and the crystallographic orientation of the copper grains was identified on an inverse pole figure (IPF) orientation map obtained by EBSD. Then, two of the essential factors of the diffusion of Cu atoms, the surface energies and the energy barriers of different crystallographic planes of Cu grains, were calculated using first principles. The CASTEP program [29] was utilized to implement the first-principles calculations based on density functional theory. Local density approximation (LDA) was used for lattice-structure optimization, in which the total energy convergence value was set as 1.0 × 10^−5^ eV/atom. In the lattice-structure optimization, the force on a single atom and stress deviation were less than 0.03 eV/Å and 0.02 GPa, respectively; the convergence precision of self-consistent field (SCF) and cut-off energies were 1.0 × 10^−6^ eV/atom and 400 eV, respectively. Considering that the surface energy and the energy barrier of a certain diffusion path were more like two essential attributes of a material, no electronic current was applied in the first-principles calculation, and a diffusion path of a Cu atom from the surface to the vacuum layer along the normal direction, which has a length of 8 Å, was set to calculate the energy barrier.

## 3. Results and Discussion

### 3.1. Realization of Crystallographic Characteristic of Cu Substrate and Cathode Dissolution

**Figure 2:**
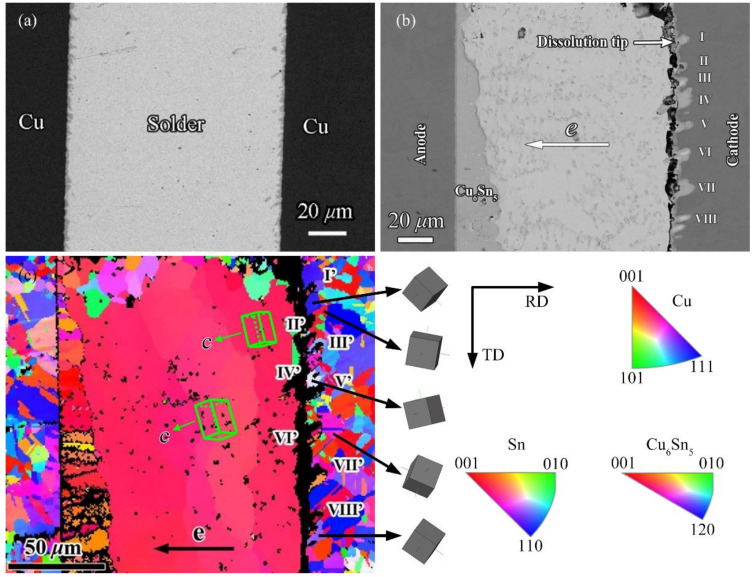
(**a**) As-reflowed Cu/SAC305/Cu joint; (**b**) a Cu/SAC305/Cu joint under the current stressing of 1.5 × 10^4^ A/cm^2^ at 125 °C for 200 h; (**c**) corresponding IPF-X color orientation maps in (**b**), I’ in (**c**) corresponding to I in (**b**), II’ to II, and so on.

**Figure 3:**
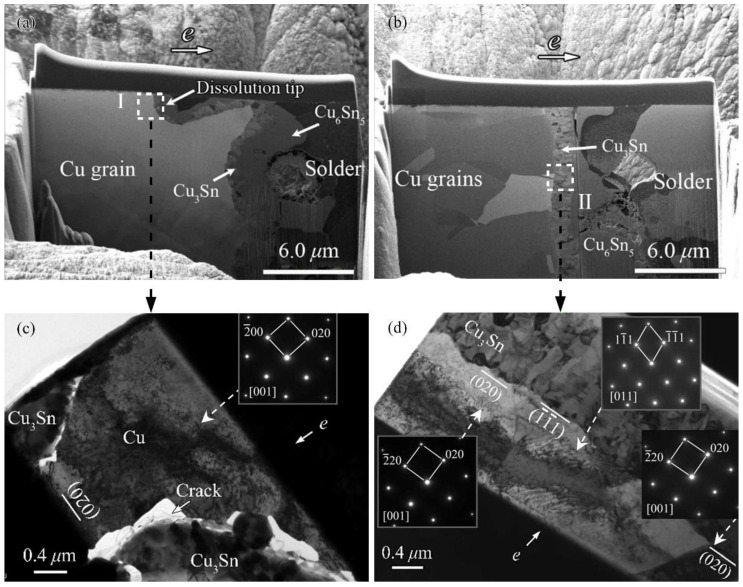
FIB/BSE images of internal cross-sectional view around the dissolution tip (**a**) and the nondissolution tip (**b**), and bright field images and selected-area diffraction patterns of copper grains adjacent to the cathodic Cu/IMC interface: (**c**) at ‘I’ in Figure 3a and (**d**) at ‘II’ in Figure 3b, with the inserts showing the diffraction patterns of the corresponding copper grains.

Figure 2a,b show the as-reflowed Cu/SAC305/Cu joint before and after EM, respectively. Obviously, a thin, scallop-like Cu_6_Sn_5_ layer forms at the two interfaces of the as-reflowed solder joint, and the average thickness of Cu_6_Sn_5_ layer is about 1.5 μm. After EM, not only does the interfacial IMC evolution exhibit the polarity effect, but cracks also form near by the cathodic interface. A few cathode dissolution tips (labeled I–VIII in Figure 2b) thrust into the Cu substrate, that is, inhomogeneous cathode serrated dissolution occurs, manifesting as uneven distances between two adjacent dissolution tips. Figure 2c presents the corresponding IPFs color orientation maps along the *x*-axis of Figure 2b. The lattice of the Sn grain is tetragonal (*a* = *b* = 5.83 Å is much larger than that of c, and c = 3.18 Å), thus Sn grain orientation can be referred to as the *c* axis of Sn grain. Generally, three large Sn grains extend from left to right in the Sn matrix of the joint, as shown in Figure 2c, in which the offset of the *c*-axis of the Sn grain near the cathode side from the current direction is approximately 18° (that is, it is nearly paralleled to the current direction). The crystallographic orientations of the Sn grains in the middle and left grains of the map are also nearly paralleled to the current direction, see the two lattices in Figure 3c, suggesting the consistent diffusion behavior of the Cu atoms in the three large Sn grains. In this case, it can be concluded that the cathodic Cu–solder interface should be approximatively planar, or the serrated dissolution tips should be homogeneous at the cathode side. Obviously, this conjecture contradicts the experimental observations (see the inhomogeneous serrated dissolution or the dissolution tips in Figure 2b). Furthermore, in the EBSD results of Figure 2c, the orientations and sizes of all Cu grains adjacent to both Cu–solder interfaces are random. For example, the five small images at the right side of Figure 2c, labeled 1–5 from top to bottom, are, respectively, aligned with the “blue”, “red”, “white”, “pink”, and “gray” copper grains in the color map. Obviously, the (111) plane of the blue copper grains (represented by grain 1) is nearly vertical to the current direction, while the planes of the other four grains, which are perpendicular to the current direction, are not the (111) plane.

Comparing the cathode dissolution tips with the characteristics of the corresponding copper grains, an interesting feature can be found: the dissolution tip seldom formed at the “blue” copper grains with the (111) plane but easily formed near other copper grains. In a previous study [30], we reported that when the (020) plane (i.e., the (001) plane) of the copper grain was nearly vertical to the current direction, the plane was easily dissolved and cathode dissolution tips formed on the Cu substrate, while the (−1−11) plane (i.e., the (111) plane) remained mostly intact, see Figure 3 [30]. It is noting that owing to the channeling effect of ion beams originating from different orientations of copper grains [31], different Cu grains with different crystallographic orientations show different contrasts after cutting by focused ion beam (FIB) and under backscattered electron (BSE) mode. Clearly, it can be concluded from Figure 2 and Figure 3 that the crystallographic characteristics of the Cu grains significantly influences the cathode dissolution behavior in the substrate.

### 3.2. Mechanism of Crystallographic Characteristic Effect of Cu Substrate on Serrated Cathode Dissolution

The IMC layer dissolves and releases Cu atoms into the SAC305 solder, meanwhile, it receives the Cu atoms released from the Cu substrate under high current stressing. Under high current stressing, Cu atoms can diffuse from Cu substrate to solder matrix via the IMC layer. When the *c*-axes of the Sn grains are parallel to the current direction, Cu atoms within them are easiest to diffuse [2,8]. As the *c*-axes of the Sn grains in the solder matrix shown in Figure 2c are nearly parallel to the current direction, the Cu atoms released from the Cu substrate can promptly diffuse to the anode side. Thus, the degree of difficulty of releasing Cu atoms from the Cu substrate critically influences the IMC formation and subsequent cathode dissolution. When the surface energy of a given crystallographic plane is higher, the atoms in the plane are easier to be released [32,33]; for this reason, investigating the differences of the surface energies and other parameters of Cu atoms in the (111) and (001) planes are vitally important. In this study, the surface energy (*E*_surf_) at a certain plane was calculated by the first-principles method based on density functional theory and implemented by the CASTEP program [29]. The calculation is given by

(1) $$E_{surf}=\frac{1}{2A}(E_{slab}-NE_{bulk}),$$

where *A* is the surface area. *E*_slab_ and *N* are the total energy and number of atoms in the slab model, respectively, and *E*_bulk_ denotes the bulk energy per atom. Table 1 lists the surface energies calculated by this model, in which the surface energies of (111) and (001) planes are 1.8077 J/m^2^ and 1.9779 J/m^2^, respectively. Clearly, the Cu atoms are easily released from the (001) plane, which possess a higher surface energy than the (111) plane.

**Figure 4:**
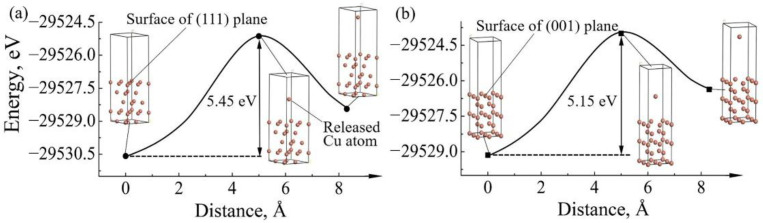
Total energy of releasing Cu atom versus the distance between the released Cu atom and the surface of a crystal plane, estimated in slab models of (**a**) the (111) plane and (**b**) the (001) plane.

After release from the crystallographic plane, the atoms must overcome the energy barrier of this plane to diffuse under current stressing. The higher the energy barrier, the more difficult the atoms releasing nearby this plane. Therefore, the energy barriers of releasing Cu atoms from the (111) and (001) planes toward phase boundaries, which can be simplified as vacuum layers, were also calculated. Figure 4 plots the total energy versus the distance between the released Cu atom and the crystal surface. The energy barriers of releasing Cu atoms from the surfaces of the (111) and (001) planes were 5.45 and 5.15 eV, respectively. Obviously, owing to the lower energy barrier, Cu atoms in the (001) plane are easier to diffuse compared to the (111) plane.

Combining with the analyzed results on the surface energies and the energies barrier of the two different crystalline planes, it can be deduced that the higher surface energy and lower energy barrier of a certain crystallographic plane are two of the essential factors of the cathode dissolution tips and the serrated cathode interfaces of Cu/SAC305/Cu solder joints during EM. Some planes of Cu grain (herein, (001) plane) in the cathodic substrate dissolve more easily than other planes (herein, (111) plane), which induces the inhomogeneous serrated cathode dissolution. Therefore, it is proposed that adjusting the copper grain to a suitable crystallographic plane or direction is a promising method to improve the EM reliability of solder joints.

## 4. Conclusions

During EM, the crystallographic characteristics of the copper grains at the cathode side of the Cu/SAC305/Cu joints significantly influences the local cathode dissolution behavior of the Cu substrate. The cathode dissolution tip is obvious at the (001) planes of copper grains oriented perpendicularly or near-perpendicularly to the current direction and minimal in the (111) plane. The different release rates of Cu atoms along the different crystallographic planes, which are affected by the different surface energies and energy barrier of the different crystallographic planes, lead to the inhomogeneous cathode dissolution of Cu substrate.

## Figures and Tables

**Table 1:** Surface energies calculated by the first-principles equation.

Plane	*A* (10^−20^ m^2^)	*E*_slab_ (eV)	*N*	*E*_bulk_ (eV)	*E*_surf_ (J/m^2^)
(111)	5.3543	−10,335.3243	7	−1476.6475	1.8077
(001)	6.1826	−10,335.0060	7	−1476.6475	1.9779

## Data Availability

The data presented in this study are available on request from the corresponding author (experimental data, W.Y; simulation data, H.Q).
